# Nonadherence to Antiepileptic Medications and Its Determinants among Epileptic Patients at the University of Gondar Referral Hospital, Gondar, Ethiopia, 2019: An Institutional-Based Cross-Sectional Study

**DOI:** 10.1155/2020/8886828

**Published:** 2020-10-28

**Authors:** Misganaw Tilahun, Netsanet Habte, Kenean Mekonnen, Mengesha Srahbzu, Daniel Ayelegne

**Affiliations:** ^1^University of Gondar Comprehensive Specialized Hospital, University of Gondar, Gondar, Amhara Region, Ethiopia; ^2^Department of Surgical Nursing, School of Nursing, College of Medicine and Health Science, University of Gondar, Gondar, Amhara Region, Ethiopia; ^3^Department of Psychiatry, University of Gondar, College of Medicine and Health Science, Gondar, Amhara Region, Ethiopia; ^4^Department of Emergency and Critical Care Nursing, School of Nursing, College of Medicine and Health Science, University of Gondar, Gondar, Amhara Region, Ethiopia; ^5^Community Health Nursing Unit, School of Nursing, College of Medicine and Health Science, University of Gondar, Gondar, Amhara Region, Ethiopia

## Abstract

**Introduction:**

Nonadherence to antiepileptic medication is the extent of a patient's passive failure to follow the prescribed therapeutic regimen. The prevalence and impact of nonadherence to antiepileptic medication are high globally. The main purpose of this study was to assess nonadherence to antiepileptic medications and its associated factors among epileptic patients at the University of Gondar Referral Hospital, Gondar, Ethiopia, 2019.

**Methods:**

An institutional-based cross-sectional study was conducted among 365 epileptic patients at the University of Gondar Referral Hospital, who were selected by a systematic random sampling technique. Data were collected by face to face interviews using a structured pretested questionnaire. Data were entered into EPI Info version 7 and then exported to SPSS version 22 for analysis. The data were described by descriptive statistics. Binary logistic regression analysis was used as a model, and variables with a *p* value of less than 0.05 were considered as statistically significant with nonadherence to antiepileptic medications.

**Results:**

A total of 356 epileptic patients participated in the study yielding a response rate of 97.5%. The overall prevalence of nonadherence to antiepileptic medications among epileptic patients attending at the University of Gondar Referral Hospital was 38.5% (95% CI: 33.1–43.8). Divorced and/or widowed marital status (AOR: 3.38 (95% CI: 1.54, 7.44)), treatment duration of 3–5 years (AOR = 3.58 (95% CI: 1.38, 9.29)), treatment duration of 5 and above years (AOR: 3.49 (95% CI: 1.53, 7.95)), comorbidity (AOR: 2.42 (95% CI: 1.08, 5.43)), side effects of antiepileptic medications (AOR: 3.36 (95% CI: 1.67, 6.74)), absence of health information (AOR: 1.98 (95% CI: 1.11, 3.52)), epilepsy-related stigma (AOR: 2.81 (95% CI: 1.57, 5.02)), and negative attitude towards antiepileptic medications (AOR: 2.46 (95% CI: 1.36, 4.45)) were significantly associated with nonadherence to antiepileptic medications.

**Conclusions:**

Prevalence of nonadherence to antiepileptic medications among epileptic patients at the University of Gondar Referral Hospital was found to be high. Hence, giving health information about epilepsy and its management will help to reduce antiepileptic medications' nonadherence.

## 1. Introduction

Epilepsy is one of the most common and widespread neurological disorders. The global burden is estimated to be 1% [1] affecting over 65 million people [2]. The burden of epilepsy is enormous particularly in the developing world that affects different dimensions including the economic expenditure in in the area of health care [1]. The World Health Organization (WHO) in 2005 reported that 80% of epileptic patients live in developing countries [1, 3].

Nonadherence to antiepileptic medication is the extent of a patient's passive failure to follow a prescribed therapeutic regimen [4]. It imposes different health consequences, including poor seizure control, increased morbidity and mortality along with the increased time of hospitalization, worsened patient outcome, poor quality of life due to enormous social stigmas, and increased health care cost [5–8]. Moreover, it also affects a person's quality of life and the family member's social, economic, and psychological health [9–12].

Despite different negative consequences, epilepsy can be controlled by medications. To have good health outcomes, adherence to antiepileptic medication is mandatory. Around 70% of people who had epilepsy are supposed to be seizure-free with optimum antiepileptic medication (AEM) treatment [13, 14]. However, in developing countries, the nonadherence of patients with chronic diseases is greater than 50%. The prevalence and impact of poor adherence in these countries are assumed to be even higher due to limited health resources and inequities in access to health care [15]. More than 30% of people with epilepsy do not attain full seizure control even with the best available treatment regimen [12]. The prevalence of AEM's nonadherence was 26% in the United States of America (USA) [10], 36.4% in the United Kingdom (UK) [16], 48.1% in China [17], 63.1% in Africa [18], and 68% in Yirgalem, Ethiopia [19].

Different studies within and across geographical areas at different times showed that some factors, like nonavailability of health information, epilepsy-related stigma, absence of social support, AEM side effects, duration of the treatment, and ways of getting the AEMs, have been associated with AEM nonadherence. On the other hand, giving brief psychoeducation about epilepsy, AEM side effects, and the importance of sticking with the recommended medication could improve AEM adherence [20]. Factors like forgetting dose, misunderstanding, unclear with clinicians' order, missing intentionally because of fear of possible side effects, and the patient's life style choice determine nonadherence to AEMs [21].

Almost all of the nonadherences to AEMs are reported to result in medical treatment failure and the reoccurrence of the seizure attack. As per the recommendation of the sustainable development goal for 2030, one of the targets is to reduce mortality from noncommunicable diseases. It is essential to study the nonadherence of AEMs and its associated factors among epileptic patients to reduce premature mortality from epilepsy.

## 2. Materials and Methods

### 2.1. Study Design, Area, and Period

An institutional-based cross-sectional study was conducted from March 10 to May 10, 2019. This study was conducted at the University of Gondar Referral Hospital. It is one of the largest referral hospitals in the Amhara Region. The University of Gondar Referral Hospital is located in Gondar town 735 km northwest of Addis Ababa. This hospital has 17 physicians, 483 nurses, 96 midwives, 78 pharmacies, and 110 laboratory technologists. This hospital serves nearly 7 million people and has 591 beds, 14 outpatient departments (OPDs), four different inpatient departments, and one emergency OPD. On average, nearly 366,408 patients visit the outpatient clinics each year, and there are more than 27,000 admissions every year. On average, the new cases of epileptic patients that visit this hospital per year were 315. This teaching hospital serves as a sole referral hospital in Northwest Ethiopia.

### 2.2. Sample Size Determination and Sampling Procedure

The sample size was determined by using a single population proportion formula. The nonadherence to antiepileptic medications proportion was taken to be 37.8% from the previous study conducted in Northwest Ethiopia [20] with a 95% confidence interval (CI) and a 4% margin of error. Since the total number of epileptic patients (*N* = 760) was less than 10,000, the correction formula was used. To compensate for the nonresponse rate, 10% of the total sample size was taken and the final sample size taken was 365.

A systematic random sampling method was used to recruit samples for the study in each day of the data collection process. The sampling fraction (*k*) was calculated by dividing the number of the study population by the sample size calculated. Thus, *K* =* N*/*n* = 760/365 = 2.08∼2. Therefore, every 2 patients were interviewed following physicians' visit. The first participant was selected by a lottery method. To avoid recycling data, special marks were used in the chart. The source populations of the study include all epileptic patients who had a follow-up visit at neurology OPD in the University of Gondar Referral Hospital. All epileptic patients who had a follow-up visit at neurology OPD in the University of Gondar Referral Hospital during the study period were considered as a study population. All adult epileptic patients who had followed treatment for at least three months at the University of Gondar Referral Hospital during the study period were included in this study. Epileptic patients who were critically ill and patients with acute psychotic illness during the study period were excluded from the study.

### 2.3. Outcome Variable

Outcome variable: the outcome variable for this study was nonadherence to antiepileptic medications. It was assessed by using the ten-item medication adherence rating scale (MARS). MARS consists of ten items with leveling of 1 = “Yes” and 0 = “No” for all items, and the total score was calculated from 10. Item of MARS was used to collect data on their adherence to antiepileptic medications and their attitude towards the medications. It was selected for its preferable quality in measuring the medication adherence in patients who are in treatment for two or more medical conditions. It also consists of questions asking adherence and drug attitude, and the total score is the sum of these questions which is believed to measure adherence in a better quality [22, 23]. Furthermore, we have preferred MARS over other screening tools because of its high reliability (Cronbach alpha = 0.76) and high internal consistency (*α* = 0.80) reported by previous study [24]. Patients who scored below 7 were considered as nonadherent to their antiepileptic medications [25].

### 2.4. Independent Variables

Sociodemographic variables include age, sex, religion, educational status, monthly income, marital status, occupation, and place of residence.

Patient-related variables include attitude towards AEMs, presence of burn marks, stigma, substance use, and forgetfulness. The standard ten-item Drug Attitude Inventory (DAI-10) scale was used to measure patients' attitudes towards antiepileptic medications; its item was a 4-point Likert scale. The mean score of attitudinal questions was calculated to categorize as having a positive attitude towards AEMs when participants scored greater than the mean score (2.90) or negative attitude towards AEMs when they scored < mean score (2.90) [26].

Perceived stigma towards epilepsy was measured by using the Kilifi stigma scale of epilepsy. A total score of 10 and above was taken to indicate the presence of perceived stigma [27].

Social life-related factor (social support): social support was assessed by using the Oslo 3-item social support scale; the first item is a 4-point Likert scale and the other two items were 5-point Likert scale. The sum score of this scale ranged from a minimum of 3 to the maximum 14. Therefore, patients who score 3–8, 9–11, and 12–14 were considered as having poor, moderate, and strong social support, respectively [28].

Clinical variables include the type of AEMs, number of AEMs, the taste of AEMs, the means of getting AEMs, side effects, comorbidity (primary psychiatric disorder, hypertension, tuberculosis, diabetes mellitus, and HIV/AIDS), duration of treatment, health information, and types of epilepsy.

### 2.5. Data Collection Procedure

Data were collected by face to face interview of antiepileptic patients by using structured and pretested questionnaire which was adopted from the literature [19, 20]. In addition to this, the questionnaire was prepared first in the English version and translated into the local language Amharic by experts' who are fluent in both languages and translated back to English to check the consistency and reliability of the translation. One master's degree holder nurse was employed as a supervisor for the recruitment and enrollment of the respondents, and three other bachelor degree nurses were employed for data collection purposes. To assure the quality of data, the pretest of the questionnaire was done on 36 epileptic patients in Felege-Hiwot Referral Hospital. Based on the pretested tools, the instrument was revised for clarity and understandability, and questions that were ambiguous and unclear were modified. One day training was provided for data collectors and supervisors on how to utilize the data collection tool. Furthermore, brief introductions about the objective of the study and unclear variables for data collectors were discussed. Data collection processes were closely followed by the principal investigator and supervisor.

### 2.6. Data Processing and Analysis

The filled questionnaire was checked for completeness of data and then entered into EPI Info version 7. To minimize data-entry errors, data were double entered and checked for discrepancies. Data analysis was carried out using the Statistical Package for Social Sciences (SPSS) software version 22. Descriptive statistics such as frequency, percentage, median, and interquartile range were employed to summarize patients' sociodemographic characteristics and other related information. The data were presented by text and tables. Binary logistic regression analysis was used to see the possible association of independent variables with nonadherence to antiepileptic medications. Independent variables with a *p* value of <0.2 in the bivariable analysis were a cut point for multivariable logistic regression analysis. Crude and adjusted odds ratios with the corresponding 95% confidence intervals were computed. Finally, those independent variables with a *p* value less than 0.05 from multivariable logistics regression were considered statistically significant.

### 2.7. Ethical Consideration

Ethical clearance was obtained from the Ethical Review Committee of the School of Nursing on behalf of the Institutional Review Board of the University of Gondar (reference number: IRB/S/N/185/03/2019). Permission was obtained from the University of Gondar Referral hospital clinical director and team leader of chronic OPD. Before data collection, written informed consent was obtained from the study participants. Each patient was informed about the objective of the study, procedures of selection, and assurance of confidentiality. Individuals were told that they had a right to withdraw from the study at any time and withdrawing may not affect the service they demanded from the hospital. Confidentiality was ensured during patient interviews. Thus, the name and address of the patient were not recorded on the data collection tool.

## 3. Results

### 3.1. Sociodemographic Characteristics of Epileptic Patients

From a total of 365 patients enrolled, 356 participants were interviewed with a response rate of 97.53%. Among the study participants, a majority (56.7%) of participants were males and the median age of participants was 27.50 years with an interquartile range of 16. Above one-third of the participants (34.6%) were in the age group between 20 and 29 years. Among the study participants, a majority of them (316, 88.8%) follow orthodox Christianity. More than half (51.4%) of the participants were single. Nearly half of the participants (164, 46.1%) were jobless, and 153 (43%) were self/private employed. More than half (55.6%) of the participants were urban residents. Around one-third of the participants (126, 35.4%) were illiterate, and 107 (30.1%) of them attended primary school. From the total of participants, 164 (46.1%) of them had less than 999 birrs monthly income (Table 1).

### 3.2. Clinical and Treatment-Related Factors

Two-third of the respondents (235, 66%) were on monotherapy. Phenobarbital (225, 63.2%) was the most commonly prescribed AEM as compared to the others. More than three-fourth of the respondents (298, 83.7%) were comfortable with the taste of AEMs. Comorbidity was reported by 53 (14.9%) of participants, and a majority of the participants (216, 60.70%) were treated for epilepsy at least for 3 years. Nearly half (44.1%) of the participants reported that they did not get health information about their condition and side effects of AEMs, and 69 (19.4%) of the participants experienced side effects of AEMs. Among the total respondents, 199 (55.9%) of them got their medication with payment. The majority of the study participants (282, 79.2%) had a generalized type of epilepsy (Table 2).

### 3.3. Patient-Related Factors

Among the total respondents, 145 (40.7%) of them skipped AEM doses. Forgetfulness (118, 81.37%) and ran out of AEMs (81, 55.86%) were the two most common reasons for skipping AEMs. Even if most of the respondents (311, 87.4%) did not use substances, alcohol (40, 11.2%) was the most commonly used substance as compared to other substances like chat (12, 3.4%) and cigarette (3, 1.7%). Nearly half (44, 12.4%) of the respondents had reported scar from falling during seizure attacks. More than one-third of the respondents (124, 34.8%) experienced perceived epilepsy-related stigma, and 165 (46.3%) of respondents had a negative attitude towards AEMs. Of the total participants, 143 (40.2%) of them got poor social support and 169 (47.5%) of respondents got moderate social support from the community (Table 3).

### 3.4. Prevalence of Nonadherence and Associated Factors of Nonadherence to AEMs

The overall prevalence of antiepileptic medication nonadherence among the study participants was 38.5% with a 95% CI (33.1, 43.8). In the final multivariable logistic regression model, independent variables that were associated with nonadherence to AEMs among epileptic patients on treatment were divorced and/or widowed marital status ((AOR): 3.38 (95% CI: 1.54, 7.44)), treatment duration of 3–5 years (AOR: 3.58 (95% CI: 1.38, 9.29)), treatment duration of above five years (AOR: 3.49 (95% CI: 1.53, 7.95)), presence of comorbidity (AOR: 2.42 (95% CI: 1.08, 5.43)), presence of antiepileptic medication side effects (AOR: 3.36 (95% CI:1.67, 6.74)), absence of getting health information from health care providers (AOR: 1.98 (95% CI: 1.11, 3.52)), perceived epilepsy-related stigma (AOR: 2.81 (95% CI: 1.57, 5.02)), and negative attitude towards AEMs (AOR: 2.46 (95% CI: 1.36, 4.45)).

The odds of being nonadherent to AEMs among divorced and/or widowed epileptic patients were 3.38 times more likely as compared to single epileptic patients. Another significant factor that was associated with nonadherence to AEMs was duration of treatment (3–5 years, 5 and above years). The odds of being nonadherent to AEMs among epileptic patients with treatment duration of 3–5 years were 3.58 times more likely as compared to epileptic patients with treatment duration of 3 months–1 year, and the odds of being nonadherent to AEMs among epileptic patients with treatment duration of above 5 years were 3.49 times more likely as compared to epileptic patients with treatment duration of 3 months to 1 year.

From clinical factors, comorbidity was significantly associated with nonadherence to antiepileptic medications. The odds of being nonadherent to AEMs among epileptic patients with comorbidity were 2.42 times more likely as compared to their counterparts.

Another clinical factor, antiepileptic medications' side effect, was significantly associated with nonadherence to antiepileptic medications. The odds of being nonadherent to AEMs among epileptic patients with antiepileptic medication side effect were 3.36 times more likely as compared to epileptic patients without antiepileptic medication side effects.

Another significant factor that was associated with nonadherence to antiepileptic medications was getting health information. The odds of being nonadherent to AEMs among epileptic patients that did not get health information were 1.98 times more likely as compared to epileptic patients who got health information.

From patient-related factors, perceived epilepsy-related stigma was significantly associated with nonadherence to antiepileptic medications. The odds of being nonadherent to AEMs among epileptic patients with perceived stigma were 2.81 times more likely as compared to epileptic patients who had no epilepsy-related stigma.

Another patient-related factor that was significantly associated with nonadherence to antiepileptic medications was negative attitude towards AEMs. The odds of being nonadherent to AEMs among epileptic patients with negative attitude towards AEMs were 2.46 times more likely as compared to epileptic patients with positive attitude towards AEMs Table(4).

## 4. Discussion

In the current study, 38.5% of patients with epilepsy were detected to have nonadhered to AEM. In this study, factors found to have significant association with nonadherence to AEMs were longer duration of illness, having medical comorbidities, developing side effects, receiving health education regarding AEMs, experiencing percieved stigma, and having poor attitude towards AEMs. This implies that a well-designed and comprehensive strategy is required to prevent the multimodal determining factors of nonadherence to AEMs among patients with epilespy. The finding of this study was consistent with the studies conducted in Jimma (41.5%) [11], Debre Markos and Finote Selam (37.8%) [20], North Carolina (39%) [29], Finland (34%) [30], and UK (36.4%) [16].

However, the finding of this study was greater than that of the studies done in USA (26%), France (21%), and India (27.7%). The probable explanation might be due to the difference in study design (retrospective cohort study design was used in USA), but the current study employed the cross-sectional study design and newer AEMs (gabapentin, lamotrigine, levetiracetam, and topiramate) which were commonly used in USA and France [10, 31, 32]; since newer medications have a lower side effect, the adherence rate could be higher. In Ethiopia, cheap and old generation antiepileptic drugs like phenobarbitone are highly used, and these might increase the nonadherence rate [33].

Nonetheless, the finding of this study was lower than that of the studies done in Yirgalem (68%) [19], Kenya (54%) [27], Nigeria (68.4%) [34], South Africa (46.4%) [35], Brazil (66.2%) [36], and China (48.1%) [17]. This difference was probably due to the difference in AEM multidrug treatment. For instance, 71.1% of people with epilepsy in Brazil, 85% in Nigeria, and 63.2% in Palestine were on multiple AEM treatment while in the current study, only 34% of people with epilepsy were in poly-AEM treatment. In addition to this, the difference might be due to the screening tool used since Morisky Medication Adherence Scale was used in Yirgalem General Hospital [19].

Regarding factors, divorced and widowed patients were about nearly three times more likely to be nonadherent as compared with those who were single in their marital status. The finding was supported by the study from Yirgalem [19]. This might be because among divorced and widowed patients, there is no support from their partners in adhering to the prescribed medication(s) and instructions given by health care professionals. In addition, most of the divorced and widowed patients were advanced in their age and more exposed to forgetfulness as compared to their counterparts [19].

Regarding the duration of treatment, those people with epilepsy who were on treatment for 5 years and above were nearly three times more likely to be nonadherent as compared to participants who were on treatment for 3 months to 1 year, and those on treatment for 3–5 years were nearly four times more likely to be nonadherent as compared to their counterparts. The present study showed that as treatment duration increases, the participants became more likely to be nonadherent, and this was supported by studies done in Jimma, Egypt, and Kenya [11, 27, 37, 38]. The possible explanation might be due to poor social support and negative attitude towards antiepileptic medications. In contrast, the current finding was not similar to the study done in India which showed that the duration of epilepsy did not have a significant association with adherence among patients with epilepsy [30]. The inconsistency in the finding might be due to the difference in the data collection methods, i.e., in India, the data were collected by using a questionnaire based on a theoretical model of compliance to calculate prevalence of nonadherence.

Participants who had comorbidity were nearly two times more likely to be nonadherent as compared to the antiepileptic patients with no comorbidity. This finding was similar to the study conducted in Yirgalem and Jimma [11, 19]. In contrast, it was not in line with the study conducted in Debre Markos and Finote Selam district hospitals [20]. The discrepancy might be due to variation of healthcare service provision.

Those participants who had AEM side effect were nearly three times more likely to be nonadherent as compared to those who had no AEM side effect. As indicated by different studies, most complaints of people with epilepsy were related to medication side effects which were probably the most common cause for discontinuing AEMs without consulting the health care giver [39]. This was similar to studies done in Debre Markos referral hospital and China which identified that patients stopped taking the drug based purely on worry of possible side effects [17, 20].

Individuals who did not get health information about their illness, duration of treatment, and drug side effect were about 1.98 times more likely to be nonadherent than their counterparts. Unless they got sufficient information, people with epilepsy tend to stop taking AEMs immediately after the seizure has been controlled or whenever they experience side effects [37].

The odds of being nonadherent for participants who had perceived epilepsy-related stigma were nearly three times as compared to those who had not. The actual physical act of having to take medication can increase the levels of stigma experienced by the person with epilepsy, and taking AEMs reminds the individuals that they have epilepsy and they may keep pill-taking in public to the minimum [21].

Individuals who had negative attitude towards the antiepileptic medications were nearly two times more likely to be nonadherent to antiepileptic medications as compared to participants who had a positive attitude. The finding was in line with the study done in China [17]. This may be due to inadequate information that patients have about their diseases and treatments which are given by health professionals.

## 5. Conclusions and Recommendations

The overall prevalence of nonadherence to antiepileptic medications among epileptic patients at the University of Gondar Comprehensive Specialized Hospital was found to be high. Marital status, duration of treatment, having comorbidity, antiepileptic medication side effects, absence of health information, epilepsy-related stigma, and negative attitude towards antiepileptic medications were statistically significantly associated with nonadherence to antiepileptic medications.

## Figures and Tables

**Table 1:** Sociodemographic characteristics of epileptic patients at UGCSH, Ethiopia, 2019 (*n* = 356).

Variables	Categories	Frequency (*n*)	Percent (%)
Age	18–20	74	20.8
	20–29	123	34.6
	30–39	76	21.3
	40–49	41	11.5
	≥50	42	11.8

Sex	Male	202	56.7
	Female	154	43.3

Marital status	Single	183	51.4
	Married	118	33.1
	Divorced	39	11.0
	Widowed	16	4.5

Religion	Orthodox	316	88.8
	Muslim	25	7.0
	Protestant	12	3.4
	Others	3	0.8

Educational status	Illiterate	126	35.4
	Primary school	107	30.1
	Secondary school	59	16.6
	Diploma and above	64	18.0

Residence	Rural	158	44.4
	Urban	198	55.6

Occupation	Jobless	164	46.1
	Self/private employed	153	43.0
	Government employee	39	11.0

Monthly income	≤999	164	46.1
	1000–1999	61	17.1
	2000–2999	48	13.5
	≥3000	83	23.3

**Table 2:** Distribution of epileptic patients by clinical and treatment-related factors at UGCSH, Ethiopia, 2019 (*n* = 356).

Variables	Categories	Frequency (*n*)	Percent (%)
Current AEM	Phenobarbital	227	63.8
	Phenytoin	144	40.4
	Sodium-valproate	38	10.7
	Carbamazepine	70	19.7
	Others	16	4.5

Comfortable with taste of AEMs	Yes	298	83.7
	No	58	16.3

Number of AEMs taken	One	235	66.0
	Two	101	28.4
	More than two	20	5.6

Type of seizure	Focal	74	20.8
	Generalized	282	79.2

Comorbidity	Yes	53	14.9
	No	303	85.1

Side effect report	Yes	69	19.4
	No	287	80.6

Duration of treatment	3 months–1 year	53	14.9
	1–3 years	87	24.4
	3–5 years	68	19.1
	>5 years	148	41.6

Getting medication	Freely	157	44.1
	On fee	199	55.9

Getting health information	Yes	199	55.9
	No	157	44.1

**Table 3:** Distribution of antiepileptic patients by patient-related factors at UGCSH, Ethiopia, 2019 (*n* = 356).

Variables	Categories	Frequency (*n*)	Percent (%)
Skipped dose	Yes	145	40.7
	No	211	59.3

Reason for skipping	Forgetfulness	118	81.37
	Ran out of medication	81	55.86
	Fear of medication side effects	20	13.79
	Feeling better	12	8.27
	Fasting	29	20.00
	Absence of AEMs	17	4.8
	Others	10	2.8

Substance use	Yes	45	12.6
	No	311	87.4

Substances used	Chat	12	3.4
	Cigarette	6	1.7
	Alcohol	40	11.2

Social support	Poor social support	143	40.2
	Moderate social support	169	47.5
	Strong social support	44	12.4

Perceived stigma	No	232	65.2
	Yes	124	34.8

Attitude to AEMs	Positive	191	53.7
	Negative	165	46.3

Presence of burn scar	No	312	87.6
	Yes	44	12.4

**Table 4:** Bivariable and multivariable analysis of factors associated with nonadherence to antiepileptic medications among epileptic patients at the University of Gondar Referral Hospital, Northwest Ethiopia, 2018 (*n* = 356).

Independent variables	Category	Nonadherence to AEMs		COR (95% CI)	AOR (95% CI)	*p* value
		No	Yes			
Marital status	Single	127	56	1	1	—
	Married	74	44	1.34 (0.82–2.19)	**1.80 (0.99–3.24)**	**0.05**
	Divorced and widowed	18	37	4.66 (2.44–8.88)	**3.38 (1.54–7.44)**	**<0.01**

Duration of treatment	3 months–1 year	40	13	1	1	—
	1–3 years	63	24	1.17 (0.53–2.56)	1.49 (0.60–3.72)	0.387
	3–5 years	39	29	2.28 (1.03–5.03)	**3.58 (1.38–9.29)**	**0.009**
	Above 5 years	77	71	2.83 (1.40–5.73)	**3.49 (1.53–7.95)**	**0.003**

Comorbidity	No	206	97	1	1	—
	Yes	13	40	6.53 (3.34–12.77)	**2.42 (1.08–5.43)**	**0.032**

Reported side effect	No	200	87	1	1	—
	Yes	19	50	6.05 (3.37–10.86)	**3.36 (1.67–6.74)**	**0.001**

Getting health information	No	70	87	3.70 (2.36–5.80)	**1.98 (1.11–3.52)**	**0.019**
	Yes	149	50	1	1	—

Perceived stigma	No	174	58	1	1	—
	Yes	45	79	5.26 (3.28–8.43)	**2.81 (1.57–5.02)**	**<0.01**

Attitude towards AEMs	Negative	70	95	4.81 (3.03–7.63)	**2.46 (1.36–4.45)**	**0.003**
	Positive	149	42	1	1	—

## Data Availability

The raw data used to support the findings of this study are available from the corresponding author upon request.
